# An abnormal course of the interazygos vein: a case report

**DOI:** 10.1186/s13256-020-02548-w

**Published:** 2020-11-30

**Authors:** Elham Shiri, Soheila Madadi

**Affiliations:** 1 Department of Anatomy, School of Medicine, Tehran University of Medical Sciences, Tehran, Iran; 2 Department of Anatomy, School of Medicine, Arak University of Medical Sciences, Arak, Iran

**Keywords:** Variation, Azygos vein, Hemiazygos vein, Accessory hemiazygos vein, Preaortic interazygos vein

## Abstract

**Background:**

The azygos venous system in the posterior mediastinum has a complex developmental pattern.

**Case presentation:**

During the dissection of this region, we encountered a variation in this system. In this case, we observed that the accessory hemiazygos and hemiazygos veins in the left side passed anterior to the aorta and drained to the azygos vein located on the left side of the vertebral column. Other structures were normal in this area.

**Conclusions:**

This variation is important in mediastinal surgery and radiographic interpretation.

## Introduction

The posterior intercostal veins drain the thoracic wall. On the two sides of the thorax, the first posterior intercostal vein directly drains into the brachiocephalic vein. The second and third and occasionally fourth intercostal veins join together to form the superior intercostal vein, which drains into the brachycephalic vein on the left side and into the azygous vein on the right side.

On the left side, the fourth or fifth to eighth intercostal veins drain into the accessory hemiazygos vein, and the three lower intercostal veins (9th to 11th) also drain into the hemiazygos vein. On the right side, the remaining posterior intercostal veins (seven or eight lower intercostal veins) drain into the azygos vein [1]. Accessory hemiazygos and hemiazygos veins pass posterior to the aorta to drain into the azygos vein. Because of the complex embryologic development of these veins, many possible variations may be expected to occur [2, 3]. The most common congenital abnormalities include agenesis of the azygos vein, azygos continuation of the inferior vena cava, an azygos lobe of the right lung, and partial venous return [4]. If one of these veins crosses the ventral aspect of the aorta, this may lead to misinterpretation of computed tomographic (CT) and magnetic resonance imaging (MRI) scans [5–8]. In the present case report, we describe a rare variation of the azygos venous system and discuss its clinical importance.

## Case presentation

**Fig. 1:**
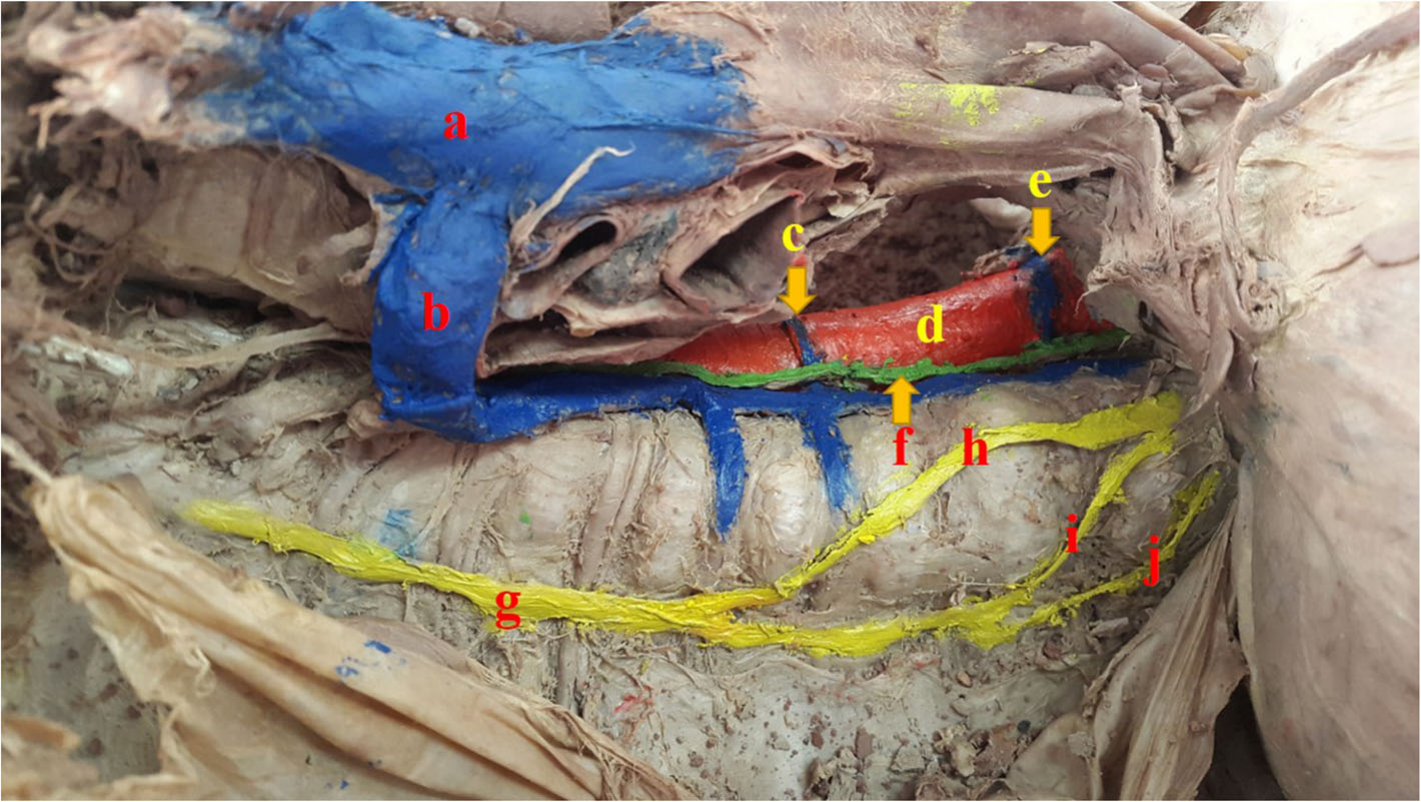
Photograph showing variations of the posterior mediastinum. **a** Superior vena cava vein. **b** Azygos vein. **c** Preaortic interazygos vein that was a direct continuation of the accessory hemiazygos vein. **d** Descending aorta. **e** Preaortic interazygos vein that was a direct continuation of the hemiazygos vein. **f** Thoracic duct. **g** Sympathetic trunk. **h** Greater splanchnic nerve. **i** Lesser splanchnic nerve. **j** Least splanchnic nerve

**Fig. 2:**
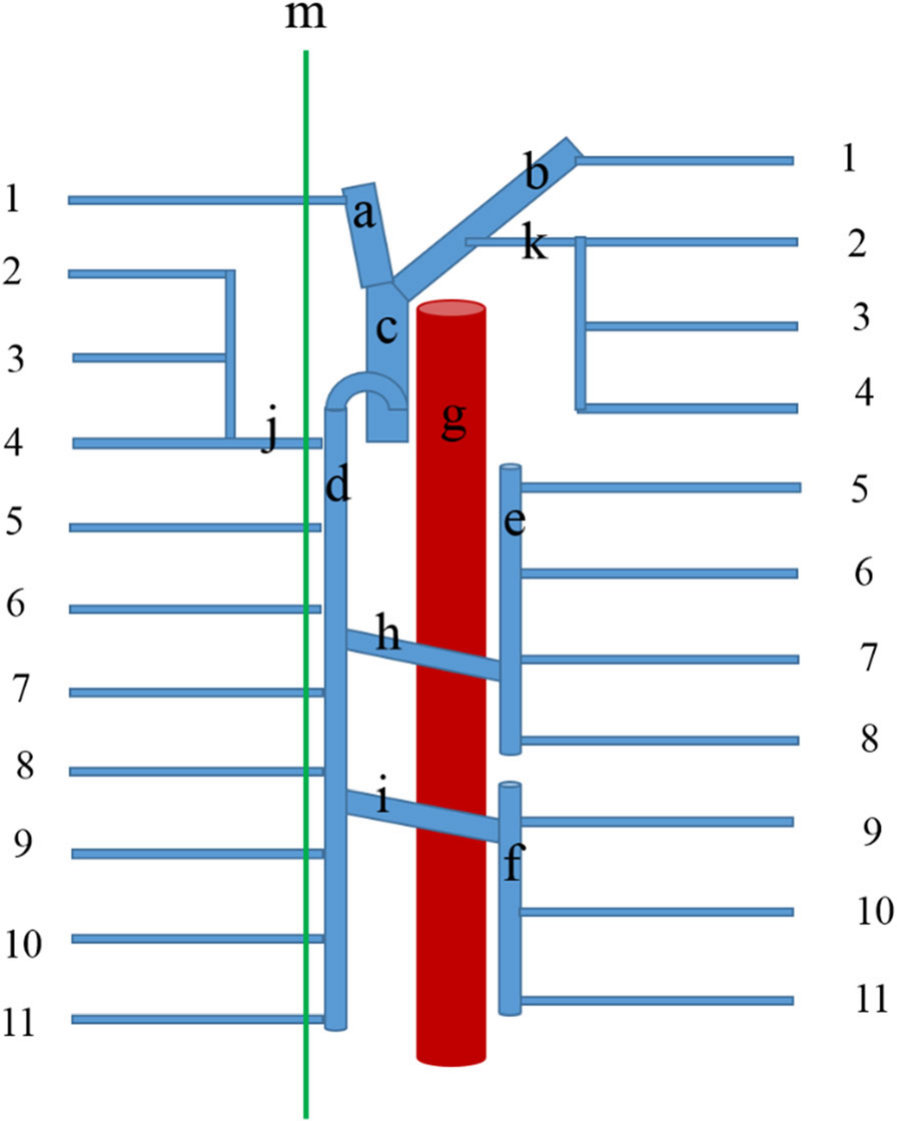
Schematic illustration of the variations. **a** Right brachiocephalic vein. **b** Left brachiocephalic vein. **c** Superior vena cava vein. **d** Azygos vein. **e** Accessory hemiazygos vein. **f** Hemiazygos vein. **g** Descending aorta. **h** Preaortic interazygos vein that was a direct continuation of the accessory hemiazygos vein. **i** Preaortic interazygos vein that was a direct continuation of the hemiazygos vein. **j** Right superior intercostal vein. **k** Left superior intercostal vein. **m** Median line

During the dissection of the thoracic part of a 70-year-old Iranian male cadaver, a variation of the azygos venous system was observed. The hemiazygos and accessory hemiazygos veins passed ventral to the aorta. These veins crossed ventral to the aorta to open into the azygos vein on the right side. The azygos vein was located on the left side of the vertebral column, then, at the top, it moved toward the right side of the vertebral column to drain into the superior vena cava vein. Also, we encountered an enlarged heart. No other abnormalities were observed. The various arrangement of these veins is shown in Figs. 1 and 2.

## Discussion

The azygos venous system consists of the azygos, hemiazygos, and accessory hemiazygos veins. This system is located on each side of the vertebral column that drains deoxygenated blood from the back and the walls of the thorax and abdomen into the superior vena cava vein.

Recent reports showed that the azygos system is very various in formation, location, and course [1, 5, 9–11]. These variations can be explained through embryological development. The azygos vein originates embryologically from the right subcardinal vein and the hemiazygos vein from the left subcardinal vein [12, 13]. Morton reported that the hemiazygos veins pass from the right side of the body and cross from the anterior aorta [7]. Sahinoglu *et al.* [8] presented a variation with absence of the right superior vena cava and the presence of a superior vena cava on the left. The right brachiocephalic vein crossed the front of the aortic arch. Keskin *et al*. [14] reported that, on the CT scan of a 52-year-old woman, the right azygos vein was present and poured into the superior vena cava. Moreover, a left azygos vein existed. It was developing the left aspect of the thoracic aorta and drained into the left subclavian vein. Instead, the left hemiazygos vein was not present. Also, there was an aneurysmatic ascending aorta that had pressured the superior vena cava.

Azygos system anomalies may be isolated or associated with other anomalies. For example, azygos continuation is common in patients with polysplenia (left isomerism) but rare in patients with asplenia and also associated with abnormal abdominal situs and a left or duplicated inferior vena cava.

In our cadaveric case, the azygos vein was on the left side of the vertebral column, and also hemiazygos and accessory hemiazygos veins were located on the left side and crossed the front of the aorta and the back of the esophagus and opened into the azygos vein at the level of the T7 and T9 vertebrae, respectively. The crossing of these structures from the front of the aorta may cause dysfunction. These variations usually occur during embryological development.

Our knowledge of the exact anatomy of the azygos system and its variations can prevent surgical mistakes. This awareness is also important in radiography because these structures may be misinterpreted as tumor, lymph node, aneurysm, iatrogenic hemorrhages, or an erroneous diagnosis in the evaluation of radiographs and MRI scans [6].

Using CT or contrast-enhanced CT techniques will help to diagnose such abnormalities. However, due to the high prevalence of variation in the azygos venous system, surgeons should pay attention to this during surgery.

## Data Availability

Not applicable.

## References

[1] Williams P, Warwick R, Dyson M, Bannister L. Gray’s anatomy. 37th ed. London: Churchill Livingstone; 1989.

[2] Williams PL. Nervous system. In: Gray’s anatomy. 38th ed. London: Churchill Livingstone; 1995. p. 1240–3.

[3] Özdemir B, Aldur M, Çelik H. Multiple variations in the azygos venous system: a preaortic interazygos vein and the absence of hemiazygos vein. Surg Radiol Anat. 2002;24(1):68–70. 10.1007/s00276-002-0008-7 12197015

[4] Donohue JR, Daly DT. Anatomy, thorax, azygos veins. In: StatPearls. Treasure Island: StatPearls Publishing; 2020. 32119317

[5] Bergman RA. Compendium of human anatomic variation: text, atlas, and world literature. Baltimore: Urban & Schwarzenberg; 1988.

[6] Celik H, Sargon M, Aldur M, Cumhur M. An anomalous course of the interazygos vein. Surg Radiol Anat. 1996;18(1):61–2. 10.1007/BF03207766 8685815

[7] Morton W. Pre-aortic drainage of the hemi-azygos veins. Report of two cases. Anat Rec. 1948;101(2):187–91. 10.1002/ar.1091010205 18863580

[8] Sahinoglu K, Cassell MD, Miyauchi R, Bergman RA. Human persistent left superior vena cava with doubled coronary sinus. Ann Anat. 1994;176(5):451–4. 10.1016/s0940-9602(11)80477-2 7978342

[9] Anson BJ. Morris human anatomy. Am J Med Sci. 1966;252(5):158.

[10] Özbek A, Dalcik C, Colak T, Dalcik H. Multiple variations of the azygos venous system. Surg Radiol Anat. 1999;21(1):83–5. 10.1007/BF01635060 10370999

[11] Grzybiak M, Szostakiewicz-Sawicka H, Treder A. Remarks on pathways of venous drainage from the left upper intercostal spaces in man. Folia Morphol (Warsz). 1975;34(3):301. 1080735

[12] Caggiati A, Barberini F. Partial agenesis of the azygos vein: a case report. Ann Anat. 1996;178(3):273–5. 10.1016/S0940-9602(96)80066-5 8712377

[13] Standring S, Ellis H, Healy J, Johnson D, Williams A, Collins P, *et al.* Gray’s anatomy: the anatomical basis of clinical practice. Am J Neuroradiol. 2005;26(10):2703.

[14] Keskin S, Keskin Z, Sekmenli N. The independent right and left azygos veins with hemiazygos absence: a rare case presentation. Case Rep Vasc Med. 2013;2013:282416. 10.1155/2013/282416 PMC3690739 23841014

